# Evidence From the China Family Panel Studies Survey on the Effect of Integrating the Basic Medical Insurance System for Urban and Rural Residents on the Health Equity of Residents: Difference-in-Differences Analysis

**DOI:** 10.2196/50622

**Published:** 2024-05-30

**Authors:** Yingying Meng, Ran Yu, Huixin Bai, Junqiang Han

**Affiliations:** 1 School of Political Science and Public Administration Center for Social Security Studies Wuhan University Wuhan China; 2 School of Public Management South-Central Minzu University Wuhan China

**Keywords:** medical insurance system integration, Urban and Rural Resident Basic Medical Insurance, URRBMI, urban and rural residents, health equity, China, difference-in-differences, DID, staggered DID

## Abstract

**Background:**

The fragmentation of the medical insurance system is a major challenge to achieving health equity. In response to this problem, the Chinese government is pushing to establish the unified Urban and Rural Resident Basic Medical Insurance (URRBMI) system by integrating the New Rural Cooperative Medical Scheme and the Urban Resident Basic Medical Insurance. By the end of 2020, URRBMI had been implemented almost entirely across China. Has URRBMI integration promoted health equity for urban and rural residents?

**Objective:**

This study aims to examine the effect of URRBMI integration on the health level of residents and whether the integration can contribute to reducing health disparities and promoting health equity.

**Methods:**

We used the staggered difference-in-differences method based on the China Family Panel Studies survey from 2014 to 2018. Our study had a nationally representative sample of 27,408 individuals from 98 cities. We chose self-rated health as the measurement of health status. In order to more accurately discern whether the sample was covered by URRBMI, we obtained the exact integration time of URRBMI according to the official documents issued by local governments. Finally, we grouped the sample by urban and rural areas, regions, and household income to examine the impact of the integration on health equity.

**Results:**

We found that overall, the URRBMI integration has improved the health level of Chinese residents (B=0.066, 95% CI 0.014-0.123; *P*=.01). In terms of health equity, the results showed that first, the integration has improved the health level of rural residents (B=0.070, 95% CI 0.012-0.128; *P*=.02), residents in western China (B=0.159, 95% CI 0.064-0.255; *P*<.001), and lower-middle-income groups (B=0.113, 95% CI 0.004-0.222, *P*=.04), so the integration has played a certain role in narrowing the health gap between urban and rural areas, different regions, and different income levels. Through further mechanism analysis, we found that the URRBMI integration reduced health inequity in China by facilitating access to higher-rated hospitals and increasing reimbursement rates for medical expenses. However, the integration did not improve the health of the central region and low-income groups, and the lack of access to health care for low-income groups was not effectively reduced.

**Conclusions:**

The role of URRBMI integration in promoting health equity among urban and rural residents was significant (*P*=.02), but in different regions and income groups, it was limited. Focusing on the rational allocation of medical resources between regions and increasing the policy tilt toward low-income groups could help improve the equity of health insurance integration.

## Introduction

### Health Inequity

With the rapid development of the global economy and medical technology, human health is improving, but health inequity is growing rather than shrinking. Health inequity in China is prominently observed between regions, urban and rural areas, and different income groups. Regional health inequity is mainly characterized by marked differences between the eastern coast and the western regions. In terms of life expectancy, the eastern provinces (or municipalities) are in the lead (eg, Beijing at 82.49 years, Shanghai at 82.55 years, Zhejiang at 80.26 years, Jiangsu at 79.32 years, and Guangdong at 79.31 years), while the western provinces exhibit lower figures, especially Qinghai (73.96 years), Yunnan (74.02 years), and the Tibet Autonomous Region (72.19 years) [1]. Health disparities also exist between urban and rural areas. From the end of 2019 to the end of 2020, the mortality rates for infants aged <1 year and children aged from 0 to 4 years in rural China were 2.40‰ and 0.78‰, respectively; the corresponding figures for urban areas were 0.89‰ and 0.25‰ [2]. Research based on China’s national microdata has proved that inequity favoring the rich for health-related quality of life remains significant in China, even after controlling for demographic factors [3].

The factors influencing health inequalities have been well discussed in previous studies. Income levels greatly influence health [4,5]. Higher-income groups not only have better access to health care but also enjoy greater health coverage [6]. In addition, there are disparities in health and the accessibility of health services for rural and susceptible populations. [7-9], with economic, medical, and educational resources being the main causes of health inequalities [10].

### Inequity in the Health Insurance System

However, relatively little attention has been paid in existing studies to health inequalities resulting from inequalities in health insurance systems. One study found that of the 5 key factors contributing to health inequities in the European region, income security and social security have the highest proportion of the burden of health inequities at 35%, including income security and social security, living conditions, social and human capital, access to health care systems and their quality, and employment and working conditions [11]. Many countries have achieved universal health insurance coverage by establishing health insurance for different groups of people [12], which can lead to fragmentation of health insurance systems, leading to inequalities in health service use and economic security [13], and this represents a major challenge to achieving health equity [14,15].

China has built the largest medical insurance network in the world, covering >1.3 billion residents but has yet to unify the system. Urban Employee Basic Medical Insurance (UEBMI) is designed to cover employed urban residents, whereas Urban Residents Basic Medical Insurance (URBMI) extends coverage to the unemployed and retired individuals, older adults, students, and children in urban areas. Meanwhile, the New Rural Cooperative Medical Scheme (NRCMS) is implemented to cater to the health care needs of rural residents. However, the 3 medical insurance systems vary in terms of coverage, fund-raising standards, security benefits, medical insurance catalogs, designated institutions, management systems, and the level of overall planning. This fragmented medical insurance system has affected the fairness of treatment access for urban and rural residents. Compared to the URBMI, the NRCMS has higher out-of-pocket medical costs but fewer drugs, services, and facilities that can be reimbursed. Urban and rural residents do not enjoy the same coverage when faced with the same medical needs. Thus, the 3 health insurance systems in China have different degrees of health promotion effects on participants. UEBMI has the greatest effect on health improvement due to the high level of security benefits. NRCMS has the least [16-18] or even insignificant effect [19]. More details about NRCMS and URBMI are provided in Table 1.

**Table 1:** Introduction to China’s basic medical insurance system (New Rural Cooperative Medical Scheme [NRCMS], Urban Resident Basic Medical Insurance [URBMI], and Urban and Rural Resident Basic Medical Insurance [URRBMI]).

	NRCMS	URBMI	URRBMI
Time	Began in 2003	Began in 2007	Some began in 2009, and most began in 2016
Coverage	Rural residents	Urban residents are not covered by the UEBMI^a^, including the unemployed, retired, older adults, students, and children	Urban and rural residents are not covered by the UEBMI
Fund-raising standards	CNY 657 (US $90.7) per person per year in 2018	CNY 776 (US $107.1) per person per year in 2018	CNY 1020 (US $140.8) per person per year in 2023
Benefits	Lower	Higher	According to high standards
Management system	National Health and Family Planning Commission of the People’s Republic of China	Ministry of Human Resources and Social Security of the People’s Republic of China	National Healthcare Security Administration, which integrates multiple functions
Level of overall planning	County-level integration	Prefecture-level integration	At the local and municipal levels, in principle, and encouragement of integration at the provincial level

^a^UEBMI: Urban Employee Basic Medical Insurance.

### Policy Background

The Chinese government is attempting to break down the fragmentation of the health insurance system and promote health equity through integrating NRCMS with URBMI. The project began as a pilot exercise in 2009 and was officially implemented in 2016. In 2016, China’s State Council issued a document named *Opinions of the State Council on Integrating the Basic Medical Insurance Systems for Urban and Rural Residents* [20], which proposed to integrate URBMI with NRCMS to establish a unified basic medical insurance system for urban and rural residents (URRBMI). The opinions clearly set out 6 integration requirements, unifying the management of 6 aspects, including coverage of systems, fund-raising policies, social security benefits, medical insurance catalogs, the management of designated institutions, and fund management. In addition, the integration as a whole follows 3 principles: regardless of whether residents belong to urban or rural areas, URRBMI will be based on a lower standard for determining contributions, a higher standard for determining treatment, and a wider standard for determining the medical insurance catalog. Overall, 6 integration requirements have enabled urban and rural residents to enjoy a fair and unified medical insurance system and reduced the cost of running medical insurance. A total of 3 principles have raised the level of treatment and narrowed the treatment gap between urban and rural residents. Through the above mentioned policy design, the integrated URRBMI has improved the fairness of urban and rural medical insurance treatment. We compared the URRBMI with NRCMS and URBMI in Table 1.

Several empirical studies have explored the impacts of URRBMI integration and found different evidence. URRBMI integration is beneficial in increasing the number of health service utilizations among rural residents [21] and in mitigating rich inequalities in outpatient benefit probabilities and benefit levels [22]. However, some research finds that the integration has a negative impact on reducing inequalities in inpatient health services [23]. Although the integration significantly increases the use of inpatient care, it has a limited impact on health outcomes for rural middle-aged and older adult residents [24].

Overall, previous studies have focused on the impact on health service use and health care costs. While a small body of literature has explored the health impacts of URRBMI integration [23,25], there is a lack of focus on health equity. The relationship between URRBMI and health equity remains uncertain. Limited health care resources in rural areas and poor areas may reduce access to health care services, while the abundance of economic and health care resources in urban areas and wealthy households may increase health care use [26], so better-off areas and households benefit more from the integration. However, the price elasticity of demand for health care services is higher in the low-income group, and the degree of response to the price of health care use changed by health care insurance will be greater, so health care insurance can promote the improvement of health care use and thus health in this group [27]. The aim of this study was to explore the differential impact of health effects across urban and rural areas, different regions, and groups of different income levels and assess whether URRBMI integration is effective in improving health equity for Chinese residents.

## Methods

### Data

This study used open data from the China Family Panel Studies (CFPS), designed by the research team at Peking University. These data are the first large-scale, comprehensive, and academically oriented social tracking survey in China. The data were made publicly available in 2010 as a baseline survey result, followed by a biennial full-sample tracking survey covering all household members in the 25 provincial-level administrative regions of mainland China [28], which represents 95% of the Chinese population. The data focus on the economic and noneconomic well-being of China’s population, including economic activity, educational attainment, family relationships and family dynamics, population migration, and physical and mental health, among many other research topics.

This study used the CFPS data from 2014, 2016, and 2018 database, which was because most cities (50/98, 51%) were consolidated in the period from 2015 to 2017. To obtain a sample suitable for the study, the raw data were further processed. At the individual level, only those individuals whose participation types were NRCMS, URBMI, or URRBMI were retained; samples with missing or abnormal information were deleted. At the city level, we deleted cities that had already achieved URRBMI integration before and in 2014, such as Changsha, Shaoguan, Yangjiang, Qianxinan Buyei and Miao Autonomous Prefecture, Dongguan, Maoming, Meizhou, Shantou, Jiangmen, Yunfu, Zibo, Qingyuan, Jieyang, Zhanjiang, Hangzhou, Chongqing, Chengdu, Wuxi, Zhuhai, and Tianjin. We also deleted cities with unclear implementation of the integration policy (judged by whether the local government has issued an official document on integrating URBMI and NRCMS). Finally, the unbalanced panel was treated into a balanced panel. Through the aforementioned processing, this study finally retained 27,408 individual samples and 98 city samples. A total of 55 (56%) city samples were in the integration group, and 43 (44%) were in the nonintegration group. The integration time of the city samples is presented in Textbox 1.

**Years the Urban and Rural Resident Basic Medical Insurance (URRBMI) was integrated in each city.**

**2015**

Ningde, Rizhao, Zaozhuang, Yantai, Ganzi Tibetan Autonomous Prefecture, Guangzhou, Laiwu, and Meishan

**2016**

Xinzhou, Ningbo, Shanghai, Ganzhou, and Yibin

**2017**

Yuncheng, Anyang, Linfen, Yongzhou, Shijiazhuang, Guangyuan, Honghe Hani and Yi Autonomous Prefecture, Pingdingshan, Jiaozuo, Fuzhou, Xinyang, Xuchang, Taiyuan, Qinhuangdao, Guilin, Zhengzhou, Hengshui, Cangzhou, Dali Bai Autonomous Prefecture, Zhumadian, Luohe, Hengyang, Xinxiang, Xingtai, Shangqiu, Xiangtan, Fangchenggang, Dingxi, Nanyang Luoyang, Luoyang, Deyang, Datong, Kaifeng, Yueyang, Loudi, Zhoukou, Yuxi, Longnan, Langfang, Ji'an, Handan, and Putian

**2018**

Baiyin, Harbin, Hegang, Lianyungang, Xiangfan, Lanzhou, Wuwei, Yichang, Wuhan, Yangzhou, Linxia Hui Autonomous Prefecture, Qingyang, Liangshan Yi Autonomous Prefecture, Luliang, Daqing, Jixi, and Daxinganling District

**2019**

Tianshui, Jinzhou, Anqing, Xuancheng, and Pingliang

**2020**

Chaoyang, Xi'an, Qiannanbu, Siping, Huludao, Pu'er, Songyuan, Qiandongnan Miao and Dong Autonomous Prefecture, Tieling, Dalian, Zunyi, Anshan, Shenyang, Dandong, Yulin, Liaoyang, Yingkou, Weinan, Fuxin, Hefei, and Tonghua

### Ethical Considerations

As a human-involved research project, CFPS regularly submits ethical reviews to the Biomedical Ethics Review Committee of Peking University and carries out data collection work upon receiving review approval (approval number IRB00001052-14010). All written informed consent forms are provided by participants aged ≥15 years or by their parents (for participants aged ≤15 years). The personal information and privacy of the interviewees are strictly protected by CFPS according to the ethics rules.

### Variables

#### Measurement of the Dependent Variable: Health Status of Residents

“Self-rated health” is the subjective evaluation of the respondents’ health status. Among many health assessment indicators, “self-rated health” is widely used in health measurement and evaluation. On the one hand, “self-rated health” takes into account the subjective knowledge of the respondents, which is scientific. On the other hand, it comprehensively evaluates the physical, psychological, and social health of individuals, so it can be more comprehensive [29]. Therefore, this study used the self-rated health score to measure the health status of residents as the dependent variable. This variable was based on the question on the CFPS questionnaire: “How would you rate your health status?” The answers were given 5 grades from “poor” to “excellent,” assigned 1 to 5 points, respectively.

#### Measurement of the Independent Variable: Whether the Sample Cities Have Integrated NRCMS With URBMI Into URRBMI

The existing research had mainly used the type of insurance participation in the questionnaire to identify the treatment group and the control group. The method of individual definition directly using the self-reported data has serious inaccuracies because ordinary rural residents are usually only concerned about whether the health insurance benefits are improved and do not care about the change of the type of insurance participation from “NRCMS” or “URBMI” to “URRBMI” because the change does not affect their medical care and reimbursement processes. Therefore, this study obtained the exact integration time of the URRBMI according to the official documents issued by local governments, such as the opinions and plans on the implementation of URRBMI integration, which was the method of “city definition.” If the integration time was earlier than the data access year t, the policy variable was assigned a value of 1; otherwise, it was assigned a value of 0. This setup produced a “treatment group” and a “control group,” as well as “pretreatment” and “posttreatment” double differences.

#### Controlled Variables

In order to alleviate the problem of missing variables, this study controlled other important variables affecting the health of residents as far as possible, according to previous relevant studies and survey data [30-32]. Controlled variables included not only the individual characteristics variables such as gender, age, marital status, type of household registration, education level, family size, annual household income per capita, and region but also the health characteristics variables such as smoking status, frequency of alcohol consumption, frequency of exercise, and chronic disease status. The definitions of the main variables and description of the mean are presented in Table 2.

**Table 2:** Definition of key variables and description of statistical analysis (N=27,408).

Variables and definition							Value		Values, mean (SD)
**Health levels**									2.8863 (1.2670)
	Poor					1			
	Fair					2			
	Good					3			
	Very good					4			
	Excellent					5			
**Policy variables** **(dummy variable)**									0.2156 (0.4113)
	The integration time is earlier than the year *t* of the data access					1			
	Otherwise					0			
**Gender** **(dummy variables)**									0.4646 (0.4988)
	Man					1			
	Woman					0			
Age in years (continuous variable)							Age of the participant		49.9984 (14.1595)
**Marital status (dummy variables)**									
	**Unmarried**								0.0462 (0.2098)
					Yes	1			
					Others	0			
	**Married or cohabiting**								0.8863 (0.3174)
					Yes	1			
					Others	0			
	**Divorced or widowed**								0.0675 (0.2509)
					Yes	1			
					Others	0			
**Household registration (dummy variables)**									0.0968 (0.2957)
	Nonagricultural household					1			
	Agricultural household					0			
**Education level (dummy variables)**									
	**Unschooled**								0.3426 (0.4746)
					Yes	1			
					Others	0			
	**Primary school**								0.2562 (0.4365)
					Yes	1			
					Others	0			
	**Middle school**								0.2871 (0.4524)
					Yes	1			
					Others	0			
	**High school and above**								0.1142 (0.3180)
					Yes	1			
					Others	0			
**Family size (members; dummy variables)**									
	**Small**								0.3398 (0.4737)
					Yes	1-3			
					Others	0			
	**Medium**								0.3773 (0.4847)
					Yes	4-5			
					Others	0			
	**Large**								0.2829 (0.4504)
					Yes	≥6			
					Others	0			
Income (in ten thousands of yuan; continuous variable)							Annual household income per capita		1.3693 (1.4146)
**Region (dummy variables)**									
	**East**								0.2851 (0.4515)
					Yes	1			
					Others	0			
	**Central**								0.3590 (0.4797)
					Yes	1			
					Others	0			
	**West**								0.3559 (0.4788)
					Yes	1			
					Others	0			
**Smoking (dummy variables)**									0.2904 (0.4540)
	Yes					1			
	No					0			
**Frequency of alcohol consumption (dummy variables)**									0.1587 (0.3654)
	Have drunk alcohol 3 times a week in the past					1			
	Otherwise					0			
**Frequency of exercise (per week, dummy variables)**									
	**<2 times**								0.6349 (0.4815)
		Yes				1			
		Otherwise				0			
	**2-4 times**								0.1210 (0.3262)
			Yes			1			
			Otherwise			0			
	**>4 times**								0.2441 (0.4296)
				Yes		1			
				Otherwise		0			
**Chronic disease (dummy variables)**									0.1969 (0.3976)
	Yes					1			
	Otherwise					0			

### Staggered Difference-in-Differences Model

On the basis of the fact that the timing of URRBMI integration varies in different regions, this study used the staggered difference-in-differences (DID) model to examine the impact of the integration on residents’ health level and equity. The basic idea of the DID model is to construct the treatment group and the control group and to identify the health level of residents before and after policy implementation. The staggered DID model is applicable to a special case of policy implementation, that is, policy implementation from the beginning of the pilot to the gradual extension. There are differences in implementation time in different regions [33]. Our specific models were as follows:


*Health_ijt_=β_0_+β_1_Policy_jt_+β_2_X_ijt_+δ_j_+λ_t_+ε_ijt_*
**(1)**

where the subscript *i* represents the individual number, *j* represents the number of the city where individuals live, and *t* represents the year of data access. *Health_ijt_* denotes the health level of individual *i* in city *j* in year *t*, and *Policy_jt_* is the policy dummy variable, which denotes whether the city *j* had implemented the policy of URRBMI integration in year *t*. *X_ijt_* denotes a set of control variables that affect the health of the population, *δ_j_* represents the city-fixed effect that did not change over time, *λ_t_* represents the time-fixed effect, and *ε_ijt_* is a random disturbance term. The estimated coefficient *β_1_* measures the effects of policy implementation.

## Results

### Regression Results of the Impact of URRBMI Integration on the Health Level of Chinese Residents

Table 3 reports the regression results of the effect of the URRBMI integration on the health level of Chinese residents.

**Table 3:** Regression results of the impact of Urban and Rural Resident Basic Medical Insurance integration on the health level of urban and rural residents.

Model	Policy variables (SE; 95% CI)	*t* test (*df*)	*P* value
DID^a,^^b^ (N=27,408)	0.066 (0.031; 0.006-0.125)	2.15 (27,291)	.03
DID^c^ (N=27,408)	0.068 (0.028; 0.014-0.123)	2.45 (27,291)	.01
PSM-DID^d^^,e^ (n=14,410)	0.090 (0.038; 0.015-0.166)	2.35 (14,293)	.02

^a^DID: difference-in-differences.

^b^City-fixed effect and time-fixed effect were controlled.

^c^City-fixed effect, time-fixed effect, and control variables were controlled.

^d^PSM-DID: propensity score matching combined with the difference-in-differences.

^e^City-fixed effect, time-fixed effect, and control variables were controlled.

We controlled for fixed effects and control variables in turn. The second row showed that the coefficient of the policy variable was 0.068, which was significant at the 5% statistical level, indicating that the URRBMI integration policy had significantly improved the health level of residents.

In addition, this study further used the propensity score matching combined with the DID method to address the endogenous issues that might arise from the URRBMI policy implementation “selection bias.” The role of the propensity score matching method is to look for the nonintegrated group whose characteristics are similar to those of the integrated group, that is, to select the nonintegrated group that is only in the range of the cosupport interval with the integrated group as the control group, to enhance comparability between integrated and nonintegrated groups. In this study, the nearest neighbor matching method was used to pair each sample in an integrated group with a sample in a nonintegrated group whose score was closest to each other, while limiting the propensity score gap to 0.01 (the caliper is not >0.01). Regression estimates were made using the matched samples, and the results are presented in the third row of Table 3. The regression coefficient was 0.090 after treatment, which was significant at the level of 5%. The regression result indicated that the conclusions above remained robust.

### The Impact of URRBMI Integration on the Health Equity of Chinese Residents

In light of the prominent health disparities observed across different regions, urban and rural areas, and various income groups in China, this study aimed to investigate the differential impact of the system integration on the health status of these subgroups. Specifically, through grouping estimations in subsequent sections, we sought to assess the extent to which policies contributed toward achieving health equity for Chinese residents.

#### Regression Results of the Impact of URRBMI Integration on Health Equity Between Urban and Rural Residents

As mentioned in the Introduction section, due to the split medical insurance system between rural and urban areas, URRBMI integration will have a different impact on rural and urban residents. This study identified the impact by dividing the sample into 2 groups: urban residents (nonagricultural households) and rural residents (agricultural households), based on the residents’ household registration.

The results showed that the integration had a significant contribution to the health of rural residents (B=0.070, 95% CI 0.012-0.128; *P*=.02), while it did not have a significant effect on urban residents (B=0.063, 95% CI –0.103 to 0.229; *P*=.46). This indicated that the integration significantly reduced health inequalities between urban and rural areas and achieved the policy objective better.

#### Regression Results of the Impact of URRBMI Integration on Health Equity in Different Regions

As mentioned in the Introduction section, due to the regional differences in economic and health care resource allocation, URRBMI integration will have a different impact in different regions. This study identified the impact by dividing the Chinese mainland into 3 major economic regions, namely, the eastern, central, and western regions, according to the division criteria of the National Bureau of Statistics of China. The eastern region includes Shanghai, Beijing, Tianjin, Shandong, Guangdong, Jiangsu, Hebei, Zhejiang, Fujian, and Liaoning; the central region includes Jilin, Anhui, Shanxi, Jiangxi, Henan, Hubei, Hunan, and Heilongjiang; and the western region includes Yunnan, Sichuan, Guangxi, Gansu, Guizhou, Chongqing, and Shaanxi. Table 4 reports the impact of URRBMI integration on regional health equity.

**Table 4:** Regression results of Urban and Rural Resident Basic Medical Insurance impact of the integration on health equity in different regions^a^.

Region	Policy variables (SE; 95% CI)	*t* test (*df*)	*P* value
East (n=7814)	0.016 (0.049; −0.081 to 0.112)	0.32 (7766)	.75
Central (n=9840)	0.090 (0.055; −0.018 to 0.199)	1.63 (9779)	.10
Western (n=9754)	0.159 (0.049; 0.064 to 0.255)	3.28 (9706)	<.001

^a^All regressions included the full set of control variables and fixed effects.

The results showed that the integration had a significant contribution to the health level of residents in the western region (B=0.159, 95% CI 0.064-0.255; *P*<.001), while the effect on the eastern (*P*=.75) and central (*P*=.10) regions was not significant. This suggested that the integration had, to some extent, alleviated health inequalities between regions, but the effect was limited.

#### Regression Results of the Impact of URRBMI Integration on Health Equity for Different Income Groups

As mentioned in the Introduction section, because different income groups have different health needs and different levels of access to health services, URRBMI integration will have a different impact for different income groups. This study analyzed the impact by dividing the entire sample into 4 groups: low-income, lower-middle-income, upper-middle-income, and high-income groups, based on annual household income per capita. Table 5 reports the impact of the integration on health equity for different income groups.

**Table 5:** Regression results of the impact of Urban and Rural Resident Basic Medical Insurance integration on health equity for different income groups^a^.

Income groups	Policy variables (SE; 95% CI)	*t* test (*df*)	*P* value
Low (n=6860)	−0.036 (0.067; −0.168 to 0.095)	−0.54 (6747)	.59
Lower-middle (n=6444)	0.113 (0.056; 0.004 to 0.222)	2.03 (6330)	.04
Upper-middle (n=6852)	0.088 (0.058; −0.024 to 0.201)	1.54 (6739)	.12
High (n=6852)	0.050 (0.052; −0.051 to 0.152)	0.97 (6737)	.33

^a^All regressions included the full set of control variables and fixed effects.

The results showed that URRBMI integration had a significant health promotion effect on the lower-middle-income groups (B=0.113, 95% CI 0.004-0.222; *P*=.04), but not on the other income groups (including low-income, upper-middle-income, and high-income groups). This indicated that URRBMI integration had, to some extent, alleviated the health inequity gap between different income groups, but the effect was limited.

### Robustness Tests

#### Counterfactual Test

To justify the common trend hypothesis in this study, we conducted a counterfactual test to ensure that there is no systematic difference in the health level of residents in the integrated and nonintegrated groups before URRBMI integration. This study drew on existing research to test the “counterfactual” hypothesis [34]. This was done by applying the baseline model (1) to the period when the region had not yet integrated medical insurance (removing data from years after the integration of the integration group), that is, introducing a new variable “whether the region was an urban and rural resident’ health insurance integration group,” and we assigned a value of 1 if the region was integrated later than the time of data access and otherwise assigned a value of 0, thus constructing a hypothetical treatment group and control group about “urban-rural resident’ health insurance integration.” Since the hypothetical “urban-rural health insurance integration” was not expected to have a significant impact on the health of urban and rural residents during this period, the hypothetical control group could be considered the “counterfactual” result of the treatment group. The results show that integration did not have a significant (B=0.017, 95% CI –0.115 to 0.149; *P*=.80) impact on the health of urban and rural residents before the implementation of the integration, indicating that the “counterfactual” hypothesis was largely satisfied.

#### Placebo Test

**Figure 1:**
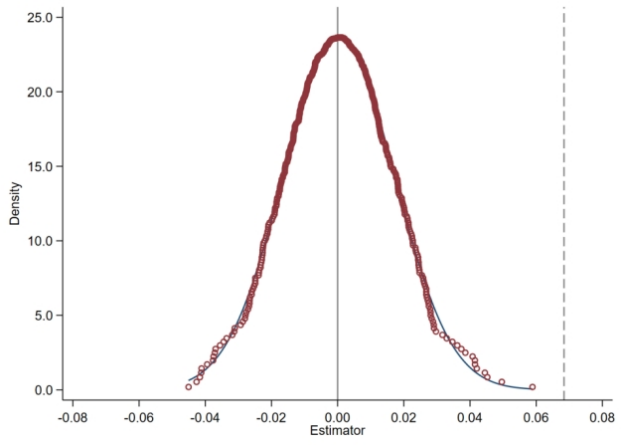
Placebo test.

In this quasi-natural experiment, the significant improvement in the self-rated health of urban and rural residents might be due to some random factors. In this study, we used some scholars’ approach to construct a placebo test to determine whether the health promotion effect of the integrated URRBMI was caused by other random factors [35]. The specific method was to use Stata software (StataCorp LLC) to make the time of the URRBMI on specific cities become random, and then repeat the random process 500 times to obtain a nuclear density distribution map of 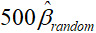, as shown in Figure 1. 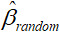 was concentrated around 0, which proved that the unobserved influence factors had little influence on the estimation results. The dotted line indicated the coefficient size of the policy variable in column 3 of Table 3. Because the dotted line was positioned far away from the distribution of the 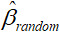, it meant that the real policy effect was different from the policy effect of the placebo test. It indicated that the coefficient of the policy variables in column 3 of Table 3 was statistically significant and stable.

#### Substitution of Variables

In addition, we conducted robustness tests by replacing variables. The ability to perform activities of daily living was chosen as a measure of objective health. This indicator measured the ability of people aged >45 years to take care of themselves. It included 7 activities in the CFPS, such as going outside for outdoor activities, eating, performing kitchen activities, taking public transportation, going shopping, cleaning, and doing laundry. If an activity could be completed independently, it was assigned a value of 1; otherwise, it was assigned a value of 0. If there were n activities that could be completed independently, the cumulative total would be n. The larger the n was, the higher the objective health level. The results show that the integration policy still produced a significant (B=0.065, 95% CI 0.015-0.115; *P*=.01) positive contribution to residents’ objective health, which indicated that the results were basically robust.

## Discussion

### Main Findings

This study used nationally representative data from China to demonstrate that URRBMI integration has a positive effect on improving health standards and promoting health equity. The results showed that URRBMI integration can improve the health level of rural residents, lower-income western regions, and lower-middle-income groups. However, the impact of reducing health disparities is still limited and has not improved the health of the central region and low-income groups. This section provides an in-depth discussion and analysis of the findings, adding additional evidence to further explain the plausibility of the findings. At the same time, this paper studied the impact path of medical insurance system integration on health, providing experience for global health insurance system reform and promoting health equity.

### The Positive Impact of the Policy

The positive health effects of medical insurance are related to policy design. First, the integration has significantly increased the level of benefits, reducing the medical burden on residents accessing health care. The reimbursement rates and cap lines of URRBMI have generally increased in all provinces and municipalities, in line with the integration principles. For example, in Shanghai, before the integration, the minimum payment rate for URBMI was 60% (for those aged <60 years), while the minimum payment rate for the NRCMS was only 50%. After the integration, the medical insurance payment rate for URRBMI was unified at 70% [36]. Second, patients’ choice of medical treatment has been increasing, which has improved the quality of medical treatment for the residents. On the one hand, the number of designated medical institutions in each province has increased after the integration. The original designated hospitals of NRCMS and URBMI have all been incorporated into the designated hospitals of URRBMI, which are located in various streets, towns, and communities (villages) in the city’s urban and rural areas, so the insured can reduce the inconvenience caused by residents seeking medical treatment in different places across counties (districts). On the other hand, the medical insurance catalog has been expanded by more than a thousand kinds of reimbursable drugs. After the integration, Inner Mongolia’s drug catalog of NRCMS has increased from 1988 to >2600, an increase of >30% [37]. Third, the process of reimbursement of medical expenses is more convenient, reducing the time and cost of medical treatment for residents. The reimbursement will be settled instantly through the social security card without the need for advance payment, eliminating all the hassles and inconveniences of medical treatment.

As mentioned in the previous paragraphs on impact pathways, rural residents have benefited more from the integration in terms of treatment levels and patients’ choice, so that URRBMI integration has promoted health equity between urban and rural residents. The increase in the drug catalog of the URRBMI is much less than that of the NRCMS, and so is the increase in the level of treatment. In addition, the quality of medical care for rural residents has improved due to the increased level of overall planning after the integration. Before the integration, URBMI was mostly coordinated at the prefectural and municipal levels, while the NRCMS was generally coordinated at the county level, so when rural residents went to a municipal hospital for medical treatment, they were seeking medical treatment in a different place, which had a lower reimbursement rate and complicated reimbursement procedures [38]. After the integration, URRBMI will in principle be coordinated at the municipal (prefectural) level and even maybe at the provincial level, breaking down the systemic and geographical differences between urban and rural residents’ access to health care and improving equity in the use of health services for rural residents [39].

### The Negative Impact of the Policy

However, the URRBMI integration has played a limited role in promoting regional health equity, mainly in that it has not contributed to the improvement of health standards in the central region. This may be related to the level of regional economic development and public health care resources. The impact of health insurance and economic factors on health has a substitution effect; that is, the health performance of medical insurance diminishes as the level of regional economic development increases [40]. Those living in regions with higher levels of economic development are likely to have less impact on health care accessibility and, thus, lesser impact on health than on factors such as income, education, and environment [41]. For those living in less developed areas, their income levels and quality of life will also be relatively low, so health insurance can significantly improve access to health care, promoting health level in poorer areas [24]. As shown in Table 6, the economic development of the eastern regions is higher than that of the central and western regions, and therefore the health promotion effect of the integration is limited for the eastern regions.

**Table 6:** Population, economic level, and health care resources in the east, central, and western regions from 2013 to 2018.

Indicators	East	Central	Western
Year-end population (×100,000)	5194.55	5391.81	3106.58
GDP^a^ per capita	79,076.79	43,555.98	42,107.50
Beds in health care facilities per 1000 population	5.03	5.41	5.61
Health technicians per 1000 population	7.51	6.24	6.89

^a^GDP: gross domestic product.

The income level of the residents in the western region is lower than that of the residents in the eastern region, and the probability of “illness without treatment” is higher. Moreover, the population in the western region is relatively small, so the per capita medical resources are relatively sufficient, as shown in Table 6. In this case, the health promotion effect of the integration is more significant (*P*<.001) in the western region, while in the central region, it may be that the reform of the system has been neglected by the policy design due to its “middle zone” of economic development and medical resources.

We also found that URRBMI integration has played a limited role in health equity for different income groups and has not improved the health of low-income groups. A number of studies have shown that there is significant heterogeneity in the health performance of health insurance according to individual income, education, age, and household registration [27,42]. The heterogeneity is particularly evident in economic and social factors [43]. In general, low-income groups are more sensitive to price changes in health services than higher-income groups [44]. However, compared with the lower-middle-income groups, the increase in funding levels brought about by health insurance consolidation has resulted in a higher financial burden of health care for the low-income groups [45], which may still discourage health needs due to the risk of poverty, leading to a lack of access to health care. To verify the this, the 2-week sickness rate and the rate of not seeking medical treatment were calculated using the questions in the CFPS questionnaire, “During the past two weeks, have you felt any physical discomfort?” and “Have you seen a doctor?” and grouped according to income. The results shows that the 2-week sickness rate is higher in the low-income group (low-income: 0.3672; lower-middle income: 0.3417; upper-middle income: 0.3141; high-income: 0.2877), and the phenomenon of not seeking medical treatment is more common (low-income: 0.8233; lower-middle income: 0.8090; upper-middle income: 0.8074; high-income: 0.7935). Therefore, the integration did not alleviate the equity of health care use among low-income groups, and the health promotion effect on low-income groups was not significant.

### Mechanism Analysis

From the regression results in the Table 3, it can be seen that the integration of URRBMI has a promoting effect on the health of rural areas, western regions, and low-income and middle-income groups; therefore, in order to further verify the mechanism of the impact of health insurance integration on health equity, we chose “choice of medical treatment” and “reimbursement ratio” as possible mechanisms to be analyzed. In the CFPS, residents’ choices of medical care included 5 categories, such as general hospitals, specialty hospitals, community health centers or township health centers, community health service stations or village health offices, and clinics, which were assigned a value of 1 to 5, respectively. The reimbursement rate was expressed as the ratio of reimbursed medical expenses to total medical expenses, where reimbursed medical expenses were equal to the difference between total medical expenses and out-of-pocket medical expenses. Table 7 reports 2 mechanisms by which health care integration affects health.

**Table 7:** Mechanism analysis: regression results of the impact of Urban and Rural Resident Basic Medical Insurance integration on the medical choices and reimbursement ratio^a^.

		β coefficient (SE)	*P* value
**Medical choices**			
	Rural	−0.156 (0.067)	.02
	Western	−0.118 (0.060)	.05
	Lower-middle	−0.036 (0.017)	.04
**Reimbursement ratio**			
	Rural	0.039 (0.014)	.004
	Western	0.036 (0.017)	.04
	Lower-middle	0.081 (0.046)	.08

^a^All regressions included the full set of control variables and fixed effects.

The first panel of Table 7 shows the regression results of the impact of the integration on the medical choices. It can be found that the coefficients of the policy variables were significant (rural: *P*=.02; western: *P*=.05; lower-middle: *P*=.04) for rural and western residents and lower-middle-income groups in the first column. This suggests that the URRBMI integration promoted the movement of residents to higher-ranking hospitals and improved the quality of care, thereby improving the health of rural and western residents and lower-middle-income groups. The second panel of Table 7 shows the regression results of the impact of the integration on the reimbursement ratio. It can be found that the reimbursement rate was expressed as the ratio of the reimbursed medical expenses to the total medical expenses, where the reimbursed medical expenses were equal to the difference between the total medical expenses and the out-of-pocket medical expenses. It can be found that the coefficients of the policy variables were significant for rural residents, western residents, and lower-middle-income groups (rural: *P*=.004; western: *P*=.04; lower-middle: *P*=.08). This suggested that the URRBMI integration increased the reimbursement rate of health insurance, which to a certain extent eased the medical burden of rural and western residents and lower-middle-income groups, thus improving the health of the population.

### Limitations

There were still several limitations to this study. Due to data limitations, we used only self-rated health as an indicator to assess the health level of the insured. Although we chose objective health to replace the dependent variable, the indicator could only measure the health of people aged >45 years, resulting in a somewhat reduced sample size. However, due to the relatively large span of the research sample, at present, China’s official data have not found relevant data to measure the health level of the whole sample of indicators. In the future, we will further seek to use a more comprehensive evaluation indicator to further test the conclusions of the study.

### Conclusions

This study found that URRBMI integration has contributed to the improvement of residents’ health, mainly due to the improvement of medical treatment and the reduction of the cost of access to health care. In terms of the impact on health equity, this paper finds that integrated URRBMI can promote health equity between urban and rural areas. However, to a limited extent, it can alleviate health disparities between regions and between different income groups. Furthermore, we also explore 2 mechanisms affecting health equity. On the basis of these findings, the policy implications of this study are as follows: first, rationally allocate medical resources among different regions. Do not neglect the development of regions with a moderate level of economic development, such as the middle region; improve the problem of insufficient resources in the middle region; and at the same time, increase support for regions with poor economic development, such as the western region. Second, the integration of medical insurance should increase the policy bias toward low-income groups to promote the use of health care services by low-income groups. This study provides experience and thoughts for China and other countries around the world in breaking down fragmented health care systems and establishing a unified health care system.

## Abbreviations

CFPS: China Family Panel Studies
DID: difference-in-differences
NRCMS: New Rural Cooperative Medical Scheme
UEBMI: Urban Employee Basic Medical Insurance
URBMI: Urban Residents Basic Medical Insurance
URRBMI: Urban and Rural Resident Basic Medical Insurance

## Data Availability

The data sets were derived from China Family Panel Studies survey from 2014 to 2018. The data used in this paper can be downloaded from the official website of the China Family Panel Studies (http://isss.pku.edu.cn/cfps/index.htm).
